# Microglia-mediated neuroinflammation and neuroplasticity after stroke

**DOI:** 10.3389/fncel.2022.980722

**Published:** 2022-08-16

**Authors:** Yuan Wang, Rehana K. Leak, Guodong Cao

**Affiliations:** ^1^Department of Neurology, University of Pittsburgh, Pittsburgh, PA, United States; ^2^Graduate School of Pharmaceutical Sciences, Duquesne University, Pittsburgh, PA, United States; ^3^Geriatric Research Education and Clinical Center, VA Pittsburgh Healthcare System, Pittsburgh, PA, United States

**Keywords:** microglia, neuroinflammation, plasticity, brain repair, ischemic stroke

## Abstract

Stroke remains a major cause of long-term disability and mortality worldwide. The immune system plays an important role in determining the condition of the brain following stroke. As the resident innate immune cells of the central nervous system, microglia are the primary responders in a defense network covering the entire brain parenchyma, and exert various functions depending on dynamic communications with neurons, astrocytes, and other neighboring cells under both physiological or pathological conditions. Microglia activation and polarization is crucial for brain damage and repair following ischemic stroke, and is considered a double-edged sword for neurological recovery. Microglia can exist in pro-inflammatory states and promote secondary brain damage, but they can also secrete anti-inflammatory cytokines and neurotrophic factors and facilitate recovery following stroke. In this review, we focus on the role and mechanisms of microglia-mediated neuroinflammation and neuroplasticity after ischemia and relevant potential microglia-based interventions for stroke therapy.

## Introduction

Microglia, the resident macrophages of the central nervous system (CNS), play vital role in CNS development, homeostasis, and disease. From development and synapse remodeling, to inflammatory insult and antigen presentation, microglia participate in various physiological processes in the brain (Wolf et al., 2017). Unlike circulating monocytes, which originate from the adult bone marrow, microglia are derived from the embryonic yolk sac and populate the CNS before its vasculogenesis (Ginhoux et al., 2010). As the most common CNS-resident innate immune cell, microglia interact directly with numerous other cell types, such as neurons, astrocytes, oligodendrocytes, and endothelial cells (Colonna and Butovsky, 2017). Under physiological conditions, resting microglia display a ramified morphology and high motility, which are favorable for immune surveillance and maintenance of homeostasis (Nayak et al., 2014). Upon detection of specific factors, such as brain injury, degeneration, or infection, microglia respond rapidly to overcome and repair CNS insults by transformation of morphology, phenotype, and function. As the primary source of proinflammatory mediators, microglia are fundamentally important for neuroinflammation and can activate and regulate a broad range of cellular responses (Salter and Beggs, 2014).

Stroke is one of the major causes of disability and mortality worldwide (Donkor, 2018). Cerebral ischemia accounts for ~85%–90% of stroke incidences (Sommer, 2017). The pathological mechanisms underlying stroke injury include energy failure, oxidative stress, disruption of the blood-brain barrier (BBB), microglial activation, inflammation, neuro-excitotoxicity, and endothelial injury (Kim et al., 2021). After an ischemic insult, microglia are activated and possess both beneficial and deleterious effects during all phases of ischemic stroke (Qin et al., 2019). Excessive activation of microglia exacerbates nerve damage and tissue injury by production of inflammatory and cytotoxic substances. However, microglia also contribute to tissue repair and functional recovery through secretion of anti-inflammatory cytokines and growth factors, clearance of cellular debris, promotion of nerve regeneration, and synapse remodeling.

Here, we briefly describe the role and mechanisms of microglia-mediated neuroinflammation and neuroplasticity after ischemic insult as well as new strategies targeting microglia for stroke treatment.

## Activation of Microglia Following Ischemic Stroke

Microglia are susceptible to ischemia, and undergo at least four morphological changes after stroke: ramified, intermediate, amoeboid, and round (Anttila et al., 2017). Ramified microglia with small cell bodies are resting microglia mainly found distal to the ischemic core or in the contralateral hemisphere. After contacting injury signals, microglia are activated and become highly motile, migrating to the lesion area, and phagocytosing cell debris or damaged neurons. Thus, intermediate activated microglia present with an enlarged cell body and short processes, and gradually develop amoeboid morphologies with very short or no processes. Intermediate and amoeboid microglia are mainly located within the peri-infarct area. Round microglia, the most activated form of microglia, are macrophage-like and can be found in the infarct core.

### Diverse phenotypes of microglia in ischemic stroke

According to the intracellular dynamics and protein production of microglia, microglia activation was previously categorized as either classical (M1) or alternative (M2). M1 activation is viewed as a proinflammatory and neurotoxic state, prompted by induction of Toll-like receptors (TLR) and interferon-γ (IFN-γ) signaling pathways. The M1 phenotype is characterized by the secretion of pro-inflammatory cytokines and chemokines, such as tumor necrosis factor-α (TNF-α), interleukin (IL)-6, IL-12, and IL-1β. M1 microglia also generate reactive oxygen species (ROS) and inducible nitric oxide synthase (iNOS), which ultimately potentiates N-methyl-D-aspartate (NMDA) receptor-mediated neurotoxicity *via* enhanced toxic effects of glutamate. Conversely, the immunosuppressive and pro-angiogenic M2 phenotype is characterized by the expression of mediators supporting anti-inflammation and tissue healing and is induced by IL-4, IL-13, and IL-10. M2 microglia not only produce anti-inflammatory cytokines, such as IL-10 and transforming growth factor β (TGF-β), but also release growth factors (i.e., insulin like growth factor 1, colony stimulating factor 1, vascular endothelial growth factor), as well as neurotrophic factors, including glial cell-derived neurotrophic factor (GDNF) and brain-derived neurotrophic factor (BDNF). Depending on different activation signals and function, M2 phenotypes are further divided into three sub-classes, including M2a, M2b, and M2c (Ma et al., 2017). M2a-like microglia, induced by IL-4 or IL-13, can inhibit inflammation and stimulate tissue repair by enhanced expression of arginase 1, CD206, and chitinase 3-like 3 (Latta et al., 2015). As an intermediate phenotype, M2b possesses the properties both of inflammatory and restorative microglia, which can by triggered by immune complexes, TLRs agonists, and IL-1R ligands (Chhor et al., 2013). Apoptotic cells, IL-10, or glucocorticoids can trigger the generation of the M2c phenotype, which is involved in tissue remodeling after the inflammatory response subsides (Mecha et al., 2015).

Despite a large body of work using the M1/M2 distinction, the M1/M2 paradigm has been inadequate to describe the full extent of microglia activation states *in vivo*, as there exist numerous overlapping functional states of microglia that lie along a continuum rather than two discrete states (Bell-Temin et al., 2015). Varying degrees of pro- and anti-inflammatory markers may even co-exist in the same cell (Yenari, 2020). Approaches such as genome-wide transcriptomics, epigenomics, and proteomics are important to facilitate a comprehensive understanding of diverse microglial profiles (Ransohoff, 2016). The recent emergence of novel single-cell techniques, such as single-cell RNA sequencing and cytometry by time-of-flight mass spectrometry (CyTOF), reveal the spatial and developmental heterogeneity of microglia (Masuda et al., 2020). A high-resolution view of the transcriptional landscape of microglia subtypes across multiple regions of CNS during development have been provided by single-cell analysis (Hammond et al., 2019; Li et al., 2019a). Neurodegenerative and neuroinflammatory diseases trigger context-specific phenotypes of microglia with diverse cellular kinetics and varying molecular characteristics (Masuda et al., 2019). Under inflammatory conditions, microglia isolated from lipopolysaccharide-injected mice showed decreased expression of homeostatic genes (*Tmem119, Siglech, P2ry12, P2ry13, Mef2c*), and upregulation of pro-inflammatory factors (Irf1, Tnf, Ccl2, and Nfkbia; Sousa et al., 2018). For example, Guo et al. suggested that *Gadd45b* in microglia, *Cyr61* in astrocytes, and *Sgk3* in oligodendrocytes play subcluster-specific roles in cell death or survival at the acute stage of ischemic stroke (Guo et al., 2021). Furthermore, in a mouse model of middle cerebral artery occlusion (MCAO), Zheng et al. reported that microglia displayed five distinct subtypes after stroke, revealing diversity in cell phenotype profiles (Zheng et al., 2022). In the aged stroke brain, Li et al. described at least six microglial subsets. According to the Li’s obserevations, MG5 is the predominant microglial phenotype in the stroke brain and displays high levels of *Top2A, Stmn1, Mki67*, and *Cdk1*, indicating a highly proliferative state and a reduction of homeostatic genes (Li et al., 2022). In contrast, MG6 was defined as the “neutrophil-like” subset, with upregulated expression of *Cxcr2, S100a8, Il1b*, and *Mmp9*, perhaps representing a stroke-specific state of microglia in the aged brain after stroke.

In recognition of the abovementioned diversities in microglia and lack of precision of the terms M1 and M2, we will cite prior research on microglial polarization with the modified terms “M1-like” and “M2-like” below.

### Signaling pathways involved in microglial activation and phenotype shifts in ischemic stroke

After an ischemic insult, intracellular molecules, including nucleic acids, lipids, and proteins, escape to the extracellular space, and have been termed danger-associated molecular patterns (DAMPs). DAMPs stimulate the pattern recognition receptors (PRRs) on microglia and induce inflammatory responses by various inflammatory signaling pathways (Dokalis and Prinz, 2019). Multiple microglial receptors, channel proteins, and enzymes participate in this process, which not only encourage microglial polarization but also serve in executing the pro-inflammatory and anti-inflammatory functions of microglia (Hou et al., 2021).

In response to different triggers, microglia exhibit heterogeneity during pathological processes, and can promote injury but also facilitate repair. The TLR4/nuclear factor kappa-B (NF-kB)/mitogen-activated protein kinase (MAPK) signaling pathway contributes to M1-like microglial activation (Zhou et al., 2019b). The interferon regulatory factor (IRF) 5-IRF4 signaling pathway also regulates microglial activation after stroke (Al Mamun et al., 2018). Overexpression of IRF-5 is associated with pro-inflammatory microglial activation, and release of IRF-4 stimulates an anti-inflammatory microglial response (Al Mamun et al., 2020). Activation of signal transducer and activator of transcription (STAT) 6 signaling axis is associated with M2-like microglial polarization, which can promote microglial efferocytosis and decrease dysfunctional phagocytosis (Cai et al., 2019). As a potential negative regulator of inflammatory mediators, phosphorylation of STAT3 is related to both proinflammatory microglial polarization and anti-inflammatory microglial polarization (Mao et al., 2021). The STAT3 signaling pathway plays a critical role in homocysteine-induced microglia activation and neuroinflammation in the rat MCAO model, and a Janus kinase (JAK)2-STAT3 inhibitor can alleviate the progression of homocysteine-associated ischemic stroke (Chen et al., 2017). In contrast, melatonin promotes microglia polarization toward an anti-inflammatory phenotype by enhancement of phosphorylated STAT3 expression in microglia (Liu et al., 2019). Furthermore, pro-inflammatory microglia can transition into anti-inflammatory microglia by various inducers (Mao et al., 2021).

### Microglia respond at different stages of ischemic stroke

In the acute phase of ischemic stroke, microglia are among the first cells to be activated and proceed to invade the peri-infarct and infarct core. Temporal analyses of microglial phenotypes in ischemic animals demonstrate that M2-like microglia were detectable at 12 h after stroke, increased in numbers within 5 days, reached a peak at days 5–7, and then fell in numbers several days after cerebral ischemia (Hu et al., 2012). During this dynamic period, M2-like microglia clear cellular debris and promote angiogenesis in the potentially salvageable penumbra. M1-like microglia were observed from day 1 post-ischemia, remained elevated from day 5 and steadily rose over 2 weeks (Jiang C. T. et al., 2020). During later stages, microglia contribute to the resolution of the infarct by phagocytosing and clearing dead cells. However, microglia may also engulf viable ischemic neurons that transiently express “eat-me” signals, and if this process is dysregulated, microglia may contribute to neuronal cell death in the peri-infarct zone (Takeda et al., 2021).

In the chronic stages of stroke, microglia can remain activated (Savitz and Cox, 2016). In animal models of stroke, delayed microglial activation has been observed in areas remote from the infarct (Walberer et al., 2014), and is believed to result in delayed and progressive neurodegeneration over time. In patients with a stroke involving the corticospinal tracts, microglial activation was observed not only in the infarct region but also in the ipsilateral thalamus and the corticospinal tract within the distant brainstem at 2 weeks post-injury. In a cortical ischemic stroke model generated by permanent MCAO, secondary thalamic injury was detected at day 7 and progressively worsened by day 28 post-stroke. Neuroinflammation with dynamic changes in microglia morphology and gene expression is considered the major physiological response during the development of secondary thalamic injury (Cao et al., 2021). At 6 months post-stroke, microglial activation is attenuated in the peri-infarct areas but persists in remote regions along the corticospinal tract (Yu et al., 2009). Chronic microglial activation may be related to myelin loss up to 1 year post-injury (Savitz and Cox, 2016). Jackson et al. (2020) found that microglia depletion mitigated long-term inflammation and enhanced white matter myelination after stroke, indicating that chronic microglial activation may be associated with delayed neurodegeneration and post-stroke cognitive impairment.

### The impact of aging on microglial response to stroke

Microglia from aged brains show alterations in cellular metabolism and energy regulation, which might evoke changes in inflammatory signaling (Flowers et al., 2017). Several immunogenic types with inflammatory and interferon-responsive profiles persist throughout the lifespan and increase with time (Hammond et al., 2019). Senescent microglia, referred to as “primed microglia,” are hypersensitive to neurotoxic and inflammatory insults, and they express high levels of pro-inflammatory mediators and reactive species, while showing deficits in phagocytosis and motility (Buga et al., 2013; Rawji et al., 2016; Wendimu and Hooks, 2022). Age exacerbates white matter damage, deteriorates functional outcomes and increases the severity of secondary neurodegeneration after stroke (Suenaga et al., 2015; Kluge et al., 2018). Suenaga et al. (2015) found that age-related deterioration in neurological function at late stages of stroke was associated with reductions in M2-like microglia. After ischemic injury, the aged brain quickly assumes an M1-like polarization state and demonstrates long-lasting impairments in M2 responses; these age-related changes are negatively linked with myelination and white matter integrity. Compared to young mice after MCAO, neovascularization was hindered in aged mice, and this hindrance was related to impaired migratory and angiogenic capacities of aged microglia. Furthermore, aged microglia exhibit lower chemoattractive abilities and reduced cell-cell interactions, which may negatively change the immune environment and slow or stymie long-term functional improvements after stroke (Jiang L. et al., 2020). Mesenchymal stromal cell-derived small extracellular vesicles (MSC-sEVs) have been shown to reduce brain macrophage infiltration into peri-infarct tissues in the aged brain. By leveraging a tolerogenic shift in immune balance, MSC-sEVs facilitate functional neurological recovery and brain tissue remodeling post-stroke in aged rats (Dumbrava et al., 2022). However, Lee et al. found that microglia depletion increases infarction volume in aged mice (Marino Lee et al., 2021). Thus, the authors suggested that even aged microglia appear to be beneficial in the early phases of ischemic stroke, despite some degree of dysregulation.

## Microglial Crosstalk with Other Cellular Brain Partners

### Microglial interactions with astrocytes in ischemic stroke

Astrocytes are the most abundant cell type in the CNS, contributing approximately 30% of brain volume (Liddelow and Barres, 2017). Astrocytes contact neurons and blood vessels simultaneously and maintain the function of neurons and the BBB (Sofroniew and Vinters, 2010). At the onset of brain injury, astrocytes become reactive and can act as a double-edged sword (Liddelow and Barres, 2017). Through release of pro-inflammatory factors and formation of glial scars, A1-like astrocytes may play a harmful role while A2-like astrocytes elicit potent neuroprotective properties by restricting inflammation and boosting tissue regeneration. For more nuanced and precise descriptions of astrocyte nomenclature, please consult the discussion of Escartin and colleagues (Escartin et al., 2021).

During CNS disease, microglia are typically activated earlier than astrocytes. By production of molecular signals, activated microglia may serve as “pioneers” and trigger reactive astrocytes, while astrocytes tend to be less responsive to injury in the absence of microglia (Chen et al., 2015). Astrocytes may serve as the “reserve forces” in the immune network and amplify the neuroinflammation cascade by their unique anatomical structures (Liu L. R. et al., 2020). Murata et al. (2020) reported that IL-1α released by microglia-derived macrophage-like cells increased astrocytic aquaporin-4 (AQP4) expression in the peri-infarct and ischemic core tissues, which ultimately exacerbated brain edema. By modulating the activation and phenotypes of microglia and astrocytes after ischemic insults, Wnt-3a has been shown to decrease infarct volumes and improve neurologic outcomes (Zhang D. et al., 2019). By inhibition of the TLR4/NF-κB pathway, cottonseed oil treatment also dampens microglial and astrocytic activation and inflammation, thereby mitigating neuronal injuries, BBB disruption, and brain edema, and reducing post-stroke neurological deficits (Liu M. et al., 2020).

**Figure 1:**
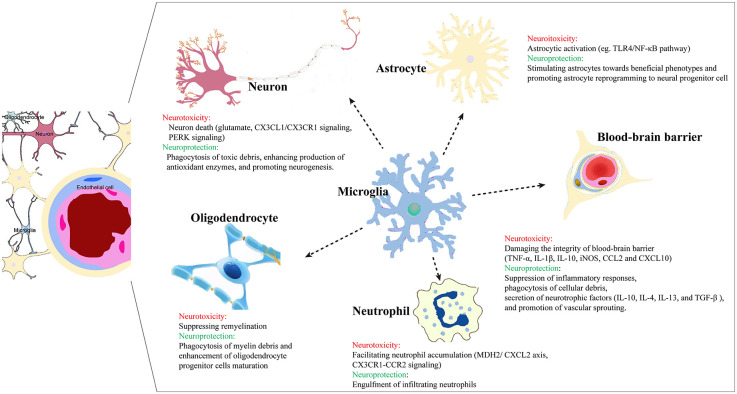
Microglia crosstalk with other cells in the brain post ischemic injury.

Through communication and cooperation, microglia and astrocytes achieve immune “optimization” in neuroinflammation. Xing et al. (2018) found that oxygen-glucose-deprived astrocytes stimulated microglia towards beneficial phenotypes that upregulated the production of IGF-1 and CD206 and promoted microglial phagocytosis, contributing to enhanced neuronal dendritogenesis. Using a mouse model of transient focal cerebral ischemia and reperfusion, Jin et al. (2017) found that microglia depletion not only promoted brain inflammation, leukocyte infiltration, and neuronal death, but also enhanced expression of inflammatory factors by astrocytes, which eventually exacerbated deficits and brain injury. Overexpression of microglia zinc finger E-box binding homeobox 1 (ZEB1) decreased the secretion of astrocytic CXCL1 through the TGF-β1 pathway, thereby attenuating neutrophil infiltration and neuroinflammation (Li et al., 2018). As stroke pathology continues to progress, the communication between microglia and astrocytes is associated with potential polarization of phenotypes, and the interaction of M2-like and A2-like glial cells may play a crucial role in neuronal survival and repair. Under a stroke-mimicking microenvironment, microglia-astrocyte cocultures increase microglial proliferation and M2-like states, while the reactive astrocytes are skewed toward the A2 type. Kim and Son (2021) demonstrated that crosstalk between astrocytes and microglia stimulated proliferation of microglia and acquisition of M2-like states in microglia and A2-like states in astrocytes *in vitro*. Cell culture studies have also shown that microglia can restrict post-ischemic astrocyte responses and confer neuroprotection. Li Z. et al. (2021) reported that small extracellular vesicles from M2-like microglia inhibited astrocyte proliferation and migration *via* the microRNA miR124 and promoted astrocyte reprogramming to neuronal progenitor cells, which eventually reduced glial scar formation and favored stroke recovery (Figure 1).

### Microglial crosstalk with neurons in ischemic stroke

After stroke onset, excitatory amino acid-mediated toxicity in neurons can induce microglia proliferation and activation and facilitate the production of pro-inflammatory mediators, which then exacerbate neuronal damage in a vicious self-amplifying cycle. Furthermore, neurons bind to microglia *via* specific receptors and activate a series of downstream signaling pathways, ultimately modulating neuron injury (Zhang Q. et al., 2021). For example, post-synaptic density 93 (PSD-93) can trigger microglial inflammatory responses through NMDA receptors in the early stages of cerebral ischemia (Zhang et al., 2015). When exposed to glutamate under injury conditions, a transmembrane chemokine specifically secreted by neurons, soluble CX3 chemokine ligand 1 (CX3CL1) disengages from the remaining structural domains and binds to its receptor CX3CR1. CX3CR1 is exclusively expressed on the surface of the microglia and serves as an activation signal (Ahn et al., 2019). Zhang et al. found that PSD-93 could bind NR2B and CX3CL1 to form a complex and increase the release of soluble CX3CL1, thereby initiating microglia activation and neuroinflammation. Specific inhibition of the PSD-93-CX3CL1 interaction may therefore attenuate neuronal cell death following a cerebral ischemia/reperfusion (I/R) insult (Zhang Q. et al., 2021). In an I/R model induced by middle cerebral artery (MCA) occlusion and reperfusion, protein tyrosine phosphatase 1B (PTP1B), which can exacerbate neuroinflammation, is highly expressed in microglia. PTP1B inhibition regulates the endoplasmic reticulum stress-autophagy axis through PERK signaling in microglia, thereby mitigating I/R-induced microglial activation and neuroinflammation, and culminating in less neuronal damage and fewer neurologic deficits after ischemia (Zhu et al., 2021).

On the other hand, microglia can also reduce neuronal cell death and infarct volume post ischemia. When exposed to harmful stimuli, neurons may secrete “help me” signals to encourage microglia to rescue neurons by improving phagocytosis of toxic debris and enhancing production of antioxidant enzymes (Suzumura, 2013). Ablation of activated microglia may therefore aggravate neuropathologic injury after ischemia. Szalay et al. found that selective removal of microglia resulted in a striking increase in infarct size, which was reversed by microglial repopulation (Szalay et al., 2016). The absence of microglia led to dysregulated neuronal responses, lack of spreading depolarization and increased excitotoxic injury, which amplified neuronal loss. On the surface, this conclusion appears to contradict Jackson and colleagues’ abovementioned finding that microglia depletion mitigates long-term inflammation (Jackson et al., 2020), but the timing and extent of the microglial depletion or other protocol differences may account for the discrepancies.

Neuroplasticity includes the spontaneous rewiring of neural networks and circuits, and functional responses in neurogenic niches. Neuroplasticity is closely associated with the dynamic evolution of activated microglia over time (Sandvig et al., 2018). Under healthy conditions, resting microglial make intimate but transient (~5 min) contacts with neuronal synapses at a frequency of approximately once per hour. These connections between microglia and synapses are dependent upon neuronal activity, and are prolonged (to about 1 h) in response to ischemia, followed by the disappearance of the injured presynaptic bouton. These findings suggest that microglia detect the functional state of synapses and determine the fate of injured axon terminals, by restoring synapse function or initiating their elimination (Wake et al., 2009). Chen et al. reported that activated microglia migrate to inhibitory synapses and displace inhibitory presynaptic terminals from cortical neurons following injury. Once inhibitory input was reduced, neurons became more excitable, contributing to increased expression of antiapoptotic and neurotrophic molecules (Chen et al., 2014). Conversely, activation of microglia complement receptor 3 (CR3) *via* nicotinamide adenine dinucleotide phosphate (NADPH) oxidase can induce long-term synaptic depression (LTD) in surrounding neurons (Zhang J. et al., 2014).

### Microglial interactions with the blood-brain barrier in ischemic stroke

The BBB is composed of a non-fenestrated monolayer of endothelial cells (ECs), linked by tight-junctions (TJs). In concert with astrocytes, pericytes, neurons, and the extracellular matrix (ECM), the BBB forms a selective physical barrier that separates the bloodstream from the brain parenchyma. Endothelia, neurons, and non-neuronal cells constitute the neurovascular unit (NVU; Zlokovic, 2008). As a crucial component of the NVU, microglia interact with the endothelium and modulate the BBB (Morrison and Filosa, 2019).

When exposed to conditioned media from oxygen-glucose-deprived ECs, ramified microglia convert into neurotoxic amoeboid shapes with increased expression of TNFα, IL-1β, IL-10, and iNOS, and decreased IGF-1 (Xing et al., 2018). As the first responder to ischemic insults, activated microglia also exert biphasic effects on the BBB, based on cellular context and the timing after stroke (Jiang et al., 2018). On one hand, microglia can phagocytose astrocytic endfeet and impair BBB integrity. On the other hand, vessel-associated microglia maintain BBB integrity *via* production of tight-junction protein Claudin-5 and physical communication with ECs (Haruwaka et al., 2019).

Yenari et al. (2006) reported that ECs tolerated oxygen-glucose deprivation (OGD)/reperfusion better than astrocytes, and that ECs showed increased vulnerability to OGD/reperfusion when cocultured with astrocytes. In addition, addition of microglia to the coculture system further aggravated the susceptibility to OGD, nearly doubling cell death and eventually potentiating damage to the BBB under ischemia-like conditions. Conversely, inhibition of microglial activation is able to improve BBB viability and integrity after ischemic stroke. Microglia can upregulate EC adhesion molecules and aggravate leukocyte infiltration by release of cytokines and chemokines (da Fonseca et al., 2014; Haley and Lawrence, 2017). Significant NVU damage may therefore unfold following exposure to IL-1β, TNF-α, and IL-6 (Ronaldson and Davis, 2020). IL-1β secreted from microglia downregulates the production of occludin, claudin-5, and zonula occludens (ZO)-1, thereby increasing BBB permeability (Kang et al., 2020). Chen et al. (2019) found that TNF-α secreted by M1-like microglia contributed to endothelial necroptosis and aggravated BBB disruption, whereas anti-TNFα treatment attenuated EC death and BBB breakdown, thereby improving post-stroke outcomes. Thurgur and Pinteaux (2019) demonstrated a close microglial-endothelium interaction in the mechanisms of IL-1/TNF-α-induced neuroinflammation. NADPH oxidase produced by microglia can induce hyperpermeability (Sumi et al., 2010) and P-glycoprotein dysfunction in ECs (Matsumoto et al., 2012), contributing to the accumulation of neurotoxic proteins in the brain. In addition, CCL2 and CXCL10 secretion by microglia can promote BBB disruption and facilitate trafficking of immune cells across the BBB (Ronaldson and Davis, 2020). Reactive oxygen species and the oxidative stress induced by microglia also aggravate endothelial cell damage and BBB dysfunction by endogenous antioxidant consumption, lipid peroxidation, DNA fragmentation, and mitochondrial failure (Thompson and Ronaldson, 2014).

Microglia also process neuroprotective properties through suppression of inflammatory responses, phagocytosis of cellular debris, and secretion of neurotrophic factors, which are related to long-term neurovascular rehabilitation and favor neurological recovery after ischemia (Yang et al., 2015). The anti-inflammatory molecules expressed by microglia (e.g., IL-10, IL-4, IL-13, and TGF-β) can limit the recruitment of neutrophils and other lymphocytes to the injury area and decrease leakage of the BBB (Kang et al., 2020). IL-10 not only exerts anti-inflammatory effects partly by downregulating NF-κB, but also protects the endothelium from oxidative stress by enhancement of antioxidant pathways and suppression of pro-oxidative reactions (Garcia et al., 2017). Ablation of microglia has been shown to decrease vascular density and exacerbate vascular permeability and hemorrhagic transformation by inhibition of TGF-β in a neonatal stroke model (Fernandez-Lopez et al., 2016). Furthermore, BDNF, endothelial growth factor (VEGF), and IL-8 produced by microglia can promote angiogenesis after injury (Jiang et al., 2018). Tian et al. (2019) found that IL4-polarized microglia can secrete exosomes containing higher amounts of miRNA-26a, resulting in increased tube formation of ECs and upregulated expression of angiogenin.

### Microglial-neutrophils interactions in ischemic stroke

After stroke, neutrophils are attracted rapidly to the injured brain, peaking in numbers between 1 and 3 days post-ischemia; this leukocyte invasion can damage the BBB integrity and exacerbate the lesion (Chu et al., 2014). Neumann et al. (2008) found that microglia protected neurons from neutrophil-mediated inflammation by engulfment of infiltrating neutrophils. Microglia depletion after stroke is associated with neutrophil accumulation and injury enlargement. Thus, microglial phagocytosis offers a fundamental defense system against neutrophil-mediated damage after stroke (Otxoa-de-Amezaga et al., 2019). On the other hand, neutrophil invasion can also stimulate interactions with microglia that aggravate ischemic injury and neurological impairments in experimental stroke (Neumann et al., 2015). By leveraging the malate dehydrogenase 2 (MDH2)/C-X-C motif ligand 2 (CXCL2) axis, long non-coding RNAs-U90926 in microglia facilitate neutrophil infiltration and aggravate ischemic brain injury (Chen et al., 2021). The CX3CR1-CCR2-dependent monocyte-microglial pathway contributes to neutrophil accumulation, neurovascular leakage, and acute ischemic injury (Faustino et al., 2019). However, the relationship between microglia and neutrophils after ischemic stroke is still incompletely understood.

### The role of microglia phagocytosis in ischemic stroke

According to “eat me” or “don’t eat me” signals on the cellular surface, microglia start or cease the process of phagocytic clearance, which is critical for neuroinflammation resolution and neuro-reorganization (Chen et al., 2022). Song et al. found that fluoxetine induces autophagy and potentiates phagocytosis in microglia, thereby exerting neuroprotection against LPS induced inflammation (Park et al., 2021). Microglia remove dead neurons and neuronal debris, which can serve as pro-inflammatory signals and disrupt CNS homeostasis (Brown, 2021). For example, the big potassium channel activator NS19504 reduced neuronal apoptosis and alleviated neurological deficits after stroke by promoting microglial phagocytosis of neuronal debris (Huang et al., 2021). Microglia, especially M2-like microglia, have the capability to phagocytose cell debris and myelin debris in ischemic stroke, attenuate the detrimental effects of inflammation, initiate synaptogenesis and neurogenesis, and sculpt a favorable microenvironment for network rewiring (Jia et al., 2021). Prefrontal stroke affects microglial phagocytic ability and presents with an accumulation of apoptotic cells in the dentate gyrus of the hippocampus (Rudolph et al., 2021). By enhancing microglial phagocytosis of myelin debris, pseudoginsenoside-F11 was found to be neuroprotective against ischemic stroke (Liu Y. et al., 2020).

Microglia also can engulf infiltrating immune cells in the injured brain and modulate their pro-inflammatory properties (Yu et al., 2022). Microglia clear granulocytes within 24 h after ischemia insults to attenuate apoptosis of neurons (Neumann et al., 2008). However, excessively activated microglia can also exacerbate neuronal loss by engulfing stressed-but-viable neurons, which hinders neurofunctional recovery and results in brain atrophy (Neher et al., 2013; Brown and Neher, 2014). Wang et al. (2021) found that ganglioside GD3 facilitated phagocytosis of microglia in the ischemic brain, thereby resulting in delayed neuronal cell death. In an ischemic stroke model, inhibition of microglial or astrocytic phagocytosis by specific deletion of MEGF10 and MERTK phagocytic receptors attenuated brain damage and improved neurobehavioral recovery (Shi X. et al., 2021). Therefore, a delicate balance between various phenotypic states is required to properly regulate neuro-inflammation and facilitate reconstruction after stroke.

### Microglial effects on neurogenesis in ischemic stroke

Early studies demonstrated that local inflammation induced by activated microglia has detrimental effects on neurogenesis. Several chemokines, such as TNF-α, IFN-γ, IL-1, and IL-6 can suppress neurogenesis and inhibit neural stem cell (NSC) survival (Sandvig et al., 2018). With the emerging complex profile of the role of microglia, microglia are now considered to exert beneficial effects on neural progenitor cell (NPC) proliferation and integration and to support functional recovery (Deierborg et al., 2010). Increased numbers of insulin-like growth factor-1 (IGF-1)-expressing microglia have been detected in the subventricular zone (SVZ) at 2, 6, and 16 weeks after stroke, which may mitigate apoptosis and increase the proliferation and differentiation of NSCs (Thored et al., 2009). The pro-neurogenic phenotype of microglia that accumulate in the SVZ offers long-term support for persistent neurogenesis after stroke. Kim et al. (2009) reported that minocycline might inhibit post-ischemic neurogenesis despite adequate suppression of inflammation, and thus showed no benefits in terms of infarct sizes or behavioral outcomes. In addition, microglia can induce non-committed oligodendrocyte progenitor cells (OPCs) to adopt a neuronal phenotype by trypsinogen secretion, thereby boosting post-insult neurogenesis (Nikolakopoulou et al., 2013).

Microglia also play a crucial role in NPC migration. Microglia can upregulate stromal cell-derived factor 1α (CDF-1α). The CDF-1α receptor CXC4 is highly expressed in NPCs, thereby driving NPC proliferation and migration towards the injury areas (Ekdahl et al., 2009). Furthermore, microglia release cytokines and growth factors that can influence the structure and activity of other cells, such as dendritic spine arborization and synaptic transmission, which serve to facilitate NPC integration into the existing circuitry (Caleo, 2015).

Raffaele et al. showed that intracerebral infusion of regenerative microglia-derived extracellular vesicles (EVs) increased protective microglia functions and prevented their senescence after ischemia. The regenerative microglia-derived EVs also favored OPC maturation and promoted neurological recovery (Raffaele et al., 2021). *In vitro* studies revealed that transmembrane TNF on EVs mediated their pro-differentiating effects on OPCs. In a MCAO animal model, endogenous OPCs proliferating into myelin-producing oligodendrocytes were found in the corpus callosum and striatum after treatment with curcumin, and this was accompanied by superior functional recovery 21 days after stroke. Ran et al. (2021) demonstrated that curcumin attenuated microglial pyroptosis through suppression of the NF-κB/NOD-like receptors containing pyrin domains (NLRP) 3 signaling pathway, thereby contributing to improvements in white matter integrity. By enhancing microglial reparative activity, osteopontin derived from brain-infiltrating Treg cells also promoted oligodendrocyte regeneration and remyelination at the chronic stages of stroke. Depletion of microglia therefore attenuated the neurorestorative effects of Treg cells on white matter (Shi L. et al., 2021).

### Microglial effects on angiogenesis in ischemic stroke

Post-stroke angiogenesis can be induced by VEGF from hypoxic tissues, and appears on the third day and persists for at least 90 days after stroke onset (Matsuo et al., 2013; Yu et al., 2021). In the acute stage of stroke, microglia contribute to the disintegration of blood vessels *via* phagocytosis of ECs and inflammatory-immunological response (Zhao et al., 2018). Selective inactivation of microglial CX3CR1 has been reported to regulate microglial migration and significantly reduce blood extravasation (Jolivel et al., 2015). By transcriptional inhibition of thioredoxin-interacting protein, energy restriction-induced sirtuin6 has been shown to suppress microglia activation and promote angiogenesis after MCAO, thereby alleviating cerebral ischemia and reperfusion (I/R)-induced injury (Song et al., 2022).

In the chronic recovery stage of stroke, microglia contribute to debris clearance and tissue repair (Ma et al., 2021). By directly producing VEGF isoforms, microglia promote vascular sprouting. By indirectly facilitating the release of VEGF-A and platelet-derived growth factor-BB from ECs, microglia also exhibit pro-angiogenic potential (Lyu et al., 2021). Fingolimod (FTY720) has been shown to display pro-angiogenic potential and to exert long-term neuroprotection after ischemic brain damage through M2-like microglial polarization (Shang et al., 2020). Post-stroke metformin or berberine treatment also facilitates angiogenesis and neurogenesis through adenosine monophosphate-activated protein kinase-dependent M2-like microglia activation, thereby improving functional recovery (Jin et al., 2014; Zhu et al., 2019).

## Microglia, A Promising Target for Neuroprotection in Ischemic Stroke

### Biochemical neuroprotectants

#### Therapeutic targets for microglia activation

Cerebral ischemia induces morphological and phenotypic changes in microglia, transforming microglia from the resting ramified phenotype into the hypertrophic phenotype (Xiong et al., 2016). An excessive activation of microglia plays a role in the neuroinflammatory response, resulting in inferior repair capacities. Thus, strategies to modulate microglia activation and phenotype are likely to be effective in controlling injury and improving brain recovery following stroke.

Colony-stimulating factor 1 receptor (CSF1R) is expressed in microglia and mainly responsible for modulating their proliferation and differentiation (Oosterhof et al., 2018). Inhibition of CSF1R has been reported to be neuroprotective in brain injury *via* minimization of microglia proliferation and activation (Henry et al., 2020; Neal et al., 2020). Ki20227, a specific inhibitor of CSF1R, can effectively protect dendritic spine densities and the dendritic structure of neurons from ischemic brain injury (Hou et al., 2020). Ki20227 downregulates the NLRP3 pathway and inflammasome activation, thereby lowering microglia numbers, attenuating microglia-related inflammation, and improving behavioral deficits (Du et al., 2020).

Naloxone, an opioid antagonist, can reduce microglia activation by inhibiting TLR signaling and reducing oxidative stress. Intermittent naloxone treatment starting on day 1 post-stroke dampened microglial activation in the striatum and thalamus and decreased neuronal death in the cortex and thalamus, resulting in behavioral recovery (Anttila et al., 2018).

G protein-coupled receptor 30 (GPR30), a novel estrogenic receptor that is highly expressed in the brain, plays an important role in the acute neuroprotective effects of estrogen (Broughton et al., 2014; Tang et al., 2014). Zhang et al. reported that GPR30 was highly expressed in microglia and significantly increased after ischemic injury. GPR30 activation relieved microglial activation and inhibited the TLR4/NF-κB pathway (Zhang et al., 2018). Administration of the GPR30 agonist G1 following ischemic injury improved neuronal survival and reduced cell apoptosis (Kosaka et al., 2012).

As the key transcription factor promoting the activation of M1-like microglia, suppression of NF-κB induces a neuroprotective phenotype in microglia (Aslanidis et al., 2015). Vx-765, a highly potent selective small molecule and BBB-permeable inhibitor of caspase-1 has also been shown to be protective in a transient MCAO model. Li et al. (2019b) found that vx-765 could not only suppress microglial activation, downregulate pro-inflammatory cytokines, and increase the production of anti-inflammatory cytokines, but also shift microglia polarization from M1 to M2-like phenotypes, an effect that was related to inhibition of NF-kB activation. Liao et al. (2020) showed that tanshinol borneol ester (DBZ) significantly decreased lipopolysaccharide (LPS)-stimulated NF-κB activation, suppressed secretion of pro-inflammatory mediators, and promoted M2-like marker expression in microglia.

The lysophosphatidic acid (LPA) pathway induces MAPK and AKT activation and phosphorylation of pro-inflammatory transcription factors (NF-κB, STAT1, and STAT3). LPA drives expression of M1-like markers and augments microglial production of reactive oxygen species and nitric oxide (NO). LPA binds and activates the lysophosphatidic acid receptor 5 (LPAR5)/protein kinase D (PKD) axis, enhancing the migratory capacity of microglia and promoting a pro-inflammatory phenotype (Plastira et al., 2017). Poly ADP-ribose polymerase 14 (PARP 14), a upstream negative regulator of LPAR5, enhances autophagy and thereby inhibits ischemia-induced microglial activation and facilitates post-stroke functional recovery (Tang et al., 2020). The latter effects are likely through the inhibition of LPAR5 by PARP 14.

#### Regulation of microglia polarizations

As microglia can assume pro-inflammatory or anti-inflammatory phenotypes and can continually switch between these states, shifting the balance toward anti-inflammatory states may be a potential strategy for tissue repair after cerebral ischemia.

Galectin-3 (Gal-3) is a β-galactoside-binding lectin and acts as an endogenous modulator of the inflammatory response. Gal-3 plays a complex and potentially time-dependent role in ischemic injury-induced microglial activation and proliferation (Lalancette-Hébert et al., 2012; Rahimian et al., 2019a). Gal-3 increases microglial ramification and mobility in an IL-4 receptor-dependent manner. Delayed treatment with recombinant Gal-3 after stroke was associated with increased chitinase-3-like protein 3 (Ym1)-positive microgliosis and decreased iNOS expression, indicating that Gal-3 shifted microglia towards anti-inflammatory phenotypes, diminished pro-inflammatory cytokine levels, and conferred neuroprotection (Rahimian et al., 2019b).

Peroxisome proliferator-activated receptor γ (PPARγ) is important in the control of inflammation and microglial phenotypes. Activation of PPARγ enhanced M2-like gene expression in microglia and promoted M2-like phenotype switching (Wang J. et al., 2018). Intravenous administration of astragaloside after cerebral I/R injury can switch microglia from M1 to M2-like phenotype in a PPARγ-dependent manner, contributing to upregulation of BDNF, IGF-1, and VEGF. These responses are associated with enhanced neurogenesis and angiogenesis (Li L. et al., 2021). The PPARγ agonist Danshensu Bingpian Zhi (DBZ) possesses anti-inflammatory and anti-oxidant activities (Liao et al., 2019). As a novel and potent regulator of glucose uptake and lipid metabolism, recombinant human fibroblast growth factor 21 (rhFGF21) also downregulated NF-κB and enhanced PPAR-γ, thereby suppressing M1-like microglia polarization and expression of pro-inflammatory factors, and favoring functional recovery in experimental stroke (Wang et al., 2020).

Adenosine monophosphate-activated protein kinase (AMPK) and nuclear factor erythroid-2-related factor-2 (Nrf2) are also involved in microglia phenotype switching. Activation of AMPK and/or inhibition of downstream mammalian target of rapamycin (mTOR) are associated with a microglial shift to the M2-phenotype and alleviation of inflammation (Li et al., 2016; Zheng et al., 2020). Annexin A1 possesses anti-inflammatory and supportive properties and can drive microglia towards the M2-like phenotype in the setting of cerebral I/R injury, through the formyl peptide receptor type 2/lipoxin A4 receptor (FPR2/ALX)-dependent AMPK-mTOR pathway. This can eventually ameliorate BBB disruption and neuronal apoptosis (Xu et al., 2021).

Nrf2 signaling not only participates in cellular defense against oxidative stress, but also negatively modulates inflammatory responses. Nrf2 plays an essential role in the regulation of ischemia-induced microglia activation (Ahmed et al., 2017), and competes with NF-κB for their common transcriptional co-activator p300, which hampers NF-κB-related inflammation (Kim et al., 2013). Moreover, antioxidant proteins such as heme oxygenase-1 (HO-1) and NAD(P)H quinone oxidoreductase 1 (NQO1) exhibit protective abilities against oxidative-stress-related neurodegeneration and inflammatory responses, and are upregulated by the Nrf2/ antioxidant response element (ARE) pathway (Kim et al., 2012). DBZ induces Nrf2 accumulation and stabilization through the Akt/glycogen synthase kinase-3β (GSK-3β)/Fyn axis, thereby contributing to antioxidant and anti-inflammatory responses in LPS-stimulated microglia (Liao et al., 2020). The novel neuroprotectant HP-1c is a hybrid of telmisartan and 2-(1-Hydroxypenty l)-benzoate (HPBA) as a ring-opening derivative of racemic 3-n-butylphthalide. HP-1c shows dual AMPK- and Nrf2-activating properties and promotes the M2-like phenotype and reduces oxidative stress, thereby alleviating ischemic cerebral injury and favoring motor recovery (Wang Y. et al., 2018).

Calycosin, a plant estrogen with antioxidant activity, preserved BDNF/tropomyosin-related kinase B signaling to attenuate inflammatory neurodegeneration and promote neuroregeneration by switching microglia from cytotoxic to neuroprotective phenotypes (Hsu et al., 2020).

#### Others

The P2Y12 receptor is a G-inhibitory-protein receptor in the platelet membrane. ADP activates the P2Y12 receptor, leading to the inhibition of adenylyl cyclase and platelet aggregation. The P2Y12 receptor is also expressed on microglia, where it is activated by ADP and participates in the pathogenesis of ischemic stroke (Li et al., 2020). Depletion of P2Y12 in microglia or inhibition of P2Y12 by the antagonist clopidogrel improved hippocampal CA1 neuron activities and limited neuronal injury after stroke (Webster et al., 2013). Ticagrelor also suppresses P2Y12-dependent signaling and reduced microglia activation and chemotaxis, and exerted neuroprotection in a MCAO model (Gelosa et al., 2014). Moreover, antagonists of P2Y12 (e.g., clopidogrel and ticagrelor) can prevent migratory and inflammatory responses in microglia (Moore et al., 2015).

Statins have been shown to be protective and anti-inflammatory in ischemic stroke (Ziedén and Olsson, 2005). Examples of statins include simvastatin and atorvastatin. Simvastatin can alter the release of cytokines and brain derived neurotrophic factor in a cholesterol-dependent manner and inhibit phagocytosis of microglia (Churchward and Todd, 2014). In a permanent MCAO mice model, Zhang et al. found that atorvastatin treatment decreased the expression of TNF-α, monocyte chemoattractant protein (MCP)-1, and IL-6 after stroke, and increased the production of IL-4. These responses were associated with higher microglial endpoints/cell and process length/cell as well as lower CD16 expression levels per cell. Atorvastatin alleviated microglia-mediated neuroinflammation by inhibiting proinflammatory polarization of microglia in the peri-infarct area, leading to better neurological function and reduced infarct volume (Zhang P. et al., 2021).

Minocycline, a broad-spectrum antibiotic, is known for its antioxidant and anti-inflammatory effects in neurological disorders (Yew et al., 2019). Several studies have reported that minocycline can modify microglial activation and promote neurovascular remodeling during stroke recovery (Yenari et al., 2006; Yang et al., 2015). Pretreatment with minocycline prevented the activation of microglia by attenuating NLRP3 inflammasome signaling, thereby ameliorating ischemia-induced brain damage (Lu et al., 2016). Minocycline can also enhance microglial M2-like polarization and suppress M1-like polarization through the STAT1/STAT6 pathway, resulting in neuronal survival and neurological functional recovery (Lu et al., 2021). Yew et al. (2019) found that early minocycline treatment decreased microglia phagocytosis and enhanced reactive astrogliosis, which influenced neuronal plasticity and contributed to the improved recovery.

Several studies have demonstrated the dynamic balance between mitochondrial fission and fusion is important during neuroinflammatory responses (Katoh et al., 2017). For example, atractylenolide III (A III) ameliorated neuroinflammation and reduced ischemia-related complications (Zhou et al., 2019a) *via* inhibition of JAK2/STAT3-dependent mitochondrial fission in microglia.

### Non-coding RNAs

Non-coding RNAs, such as micro-RNA (miRNA) and long non-coding RNA (lncRNA), are a subset of functional RNAs that participate in a variety of physiological and pathophysiological processes (Wang S. W. et al., 2018). miRNA-124, the most abundant miRNA in the CNS, regulates microglia activation and polarization, contributing to neuronal survival by stimulating trophic factors (Hamzei Taj et al., 2016a, b). In addition, lncRNA 1810034E14Rik inhibited microglial activation and downregulated the expression of inflammatory cytokines *via* suppression of the NF-κB pathway (Zhang X. et al., 2019).

### Cell-based therapy

Cell-based therapies can improve neuroplasticity, reestablish the architecture and physiology of brain tissue, and promote neurological recovery after stroke. Cell replacement, activation of endogenous pro-survival processes, nutritional support, and immunoregulation contribute to these therapeutic effects (Lovatel et al., 2014).

Mesenchymal stem cells (MSCs) are adult stem cells with the potential to differentiate into cells of the mesenchymal lineage. MSCs possess anti-inflammatory and immunomodulating properties (Abumaree et al., 2012). Intravenous administration of human umbilical cord-derived MSCs (hUC-MSCs) at 24 h after MCAO preserved adult newborn neurons and reduced neurological impairments (Lin et al., 2017). One possible mechanism underlying these positive effects is that hUC-MSCs decrease the number of hypertrophic microglia. Microglia modulate MAPK kinase/extracellular signal-regulated kinases (ERK) and phosphoinositide-3-kinase/AKT signaling pathways, thereby stimulating bone marrow MSCs (BM-MSCs) to produce GDNF and protect neurons against ischemic injury (Lv et al., 2016). Furthermore, BM-MSCs induce activation of M2-like neuroprotective microglia (Ohtaki et al., 2008). MSCs from adipose tissue (AD-MSCs) can also convert detrimental microglia phenotypes into neuroprotective phenotypes, as identified by a reduction in the expression of pro-inflammatory factors and an induction of neuroprotective and anti-inflammatory markers, of which Nrf2/HO-1 signaling and TNF-stimulated gene 6 protein in AD-MSCs seem to play a crucial role (Neubrand et al., 2014; Jha et al., 2019; Huang et al., 2020). Furthermore, Hamzei Taj et al. demonstrated that MSC-based delivery of IL13 can polarize microglia to a neuroprotective M2-like phenotype during the pro-inflammatory status of ischemic stroke (Hamzei Taj et al., 2018).

Hematopoietic stem cells (HSCs) are mainly collected from the bone marrow and can develop into mature blood cells. HSCs can also differentiate into neurons and microglia in both *in vitro* and *in vivo* studies (Wang et al., 2017). In an experimental model, bone marrow-derived mononuclear cells (MNCs) not only exerted direct neuroprotection by anti-apoptotic and anti-oxidative mechanisms, but also indirectly provided neuroprotective effects by attenuating the toxicity of activated microglia (Sharma et al., 2010).

Stem cells can secrete multiple biologically active molecules through paracrine action to participate in inflammation, angiogenesis, and regeneration (Gnecchi et al., 2016); thus, stem cell-conditioned media can exert complex positive effects under various pathological conditions of the CNS (Pawitan, 2014; Aboutaleb et al., 2019). Salikhova et al. found that human induced pluripotent stem cells (hiPSCs)-derived glial progenitor cells (GPCs)-conditioned media produces a higher content of neurotrophins, such as BDNF, GDNF, nerve growth factor (NGF), and ciliary neurotrophic factor (CNTF). In an ischemic stroke model, intra-arterial administration of GPCs-conditioned medium decreased microglia infiltration, alleviated apoptosis and inflammation, and promoted the formation of blood vessels within the damaged area (Salikhova et al., 2021).

Exosomes are secreted from hUC-MSCs and are one of the key mediators of MSC paracrine signaling. Exosomes from hUC-MSCs have been shown to repress I/R-induced pyroptosis in microglia and mitigate subsequent neuronal injury (Hu et al., 2021) *via* enhancement of FOXO3a-dependent mitophagy. AD-MSCs and BM-MSCs-derived exosomes can communicate with microglia, alter the morphology of microglia and attenuate microglia pro-inflammatory activation, consequently alleviating neuroinflammation and improving functional recovery (Sanchez-Castillo et al., 2022). Zhao et al. (2020) found that exosomes from MSCs overexpress miR-223-3p, which promoted M2 microglia transformation *via* inhibition of the cysteinyl leukotriene receptor 2 (CysLT2R) pathway.

### Non-pharmaceutical therapies

#### Hypothermia

Growing evidence shows that therapeutic hypothermia (TH) is a potentially effective treatment for stroke and can mitigate brain injury through suppression of multiple pathways, including oxidative stress, inflammatory responses, cell death signals and metabolic disruption (Wang et al., 2016). In the ischemic brain and in cortical neuronal/BV2 microglia cultures subjected to oxygen glucose deprivation (OGD), TH reduced the release of MCP-1 and macrophage inflammatory protein-1α (MIP-1α), two important mediators of microglia activation and infiltration. Microglia migration was decreased 16 h after OGD, and microglia activation in the penumbra reached a peak level at the seventh day after ischemia. These effects were attenuated by TH therapy (Lee et al., 2016).

In a transient cerebral ischemic model, hypothermia reduced the number of M1-like microglia and increased the number of M2-like microglia. Furthermore, M1-like markers were decreased at the transcriptional level after hypothermia, whereas the expression of mRNA for M2-like markers were increased. Thus, hypothermia is protective following ischemic stroke and can reduce brain tissue loss (Liu et al., 2018). Lee et al. (2016) demonstrated that TH treatment can attenuate inflammatory responses, regulate microglia activation, polarization and migration, thereby improving functional recovery after stroke.

#### Ischemic/hypoxic conditioning

Ischemic/hypoxic conditioning refers to a naturally protective but transient mechanism induced by subtoxic and brief insults, which can protect cells or tissues from a subsequent, more severe ischemic and hypoxic injury (Wang et al., 2016). The mechanisms underlying ischemic/hypoxic resistance are complex, including reinforcement of the neurovascular network, inhibition of oxidative stress reactions, regulation of autophagy, and mitigation of mitochondrial injury (Zhou et al., 2018). Previous studies have shown that conditioning therapeutics can elicit neuroprotective effects by suppressing inflammatory signals, such as TNF-α and NF-κB (Sprick et al., 2019; Rabenstein et al., 2020; Yao et al., 2020). As multiple signaling pathways targeting TLRs in ischemic conditioning converge upon microglial Type I interferon response, microglia play a crucial role in ischemic conditioning-mediated neuronal and axonal protection (McDonough and Weinstein, 2020). Studies focusing on cascades downstream of preconditioning stimuli show that numerous microglia-mediated reactions and cell-cell crosstalk take part in the regulation of the CNS response to subsequent prolonged ischemia (Gesuete et al., 2012; Benarroch, 2013). Microglia leverage TLRs and Type I IFN signaling to mediate the response to DAMPs and cytokines induced by ischemic conditioning stimuli; the conditioned microglia then upregulate Type I interferon stimulated genes, and undergo proliferation (McDonough et al., 2017). Consequentially, conditioned microglia shift to protective phenotypes and support neighboring astrocytes, neurons, and oligodendrocytes, contribute to BBB maintenance, increase neuron and oligodendrocyte survival, improve axonal integrity, and engage peripheral immune responses (McDonough and Weinstein, 2016). Intra-arterial administration of microglia preconditioned *in vitro* by optimal durations of OGD promoted functional recovery after stroke, with increased expression of remodeling factors that contribute to angiogenesis and axonal outgrowth (Kanazawa et al., 2017). EVs from OGD-preconditioned microglia stimulated M2-like switching of microglia within the ischemic cerebral environment *via* the TGF-β/Smad2/3 pathway, eventually enhancing angiogenesis and alleviating apoptosis (Zhang L. et al., 2021).

Hypoxic preconditioning-induced Nrf2 activation occurs in a GSK-3β phosphorylation-dependent manner and enhances anti-inflammatory microglia phenotype polarization after cerebral I/R, thus favoring ischemic tolerance and protective processes (Cai et al., 2021). Furthermore, ischemic/hypoxic conditioning therapeutics can influence microglia dynamics, including migration, phagocytosis, and proliferation at early stages after brain ischemia and reperfusion, promoting brain repair (Dang et al., 2018). Tantingco and Ryou (2020) found that intermittent hypoxic training can protect microglia from OGD and oxidative stress, regulate microglial polarization and increase phagocytic activity, thereby encouraging long-term recovery from ischemic stroke.

Post-conditioning involves application of the conditioning stimulus after onset of the injury, and promotes the release of VEGF from ischemia-injured neurons as a “help-me” signal that regulates microglia polarization into potentially beneficial phenotypes (Esposito et al., 2018). Xue et al. (2021) reported that post-conditioning can mitigate microglial shifts towards neurotoxic phenotypes and attenuate neuronal apoptosis and myelin damage after hypoxic-ischemic brain injury by modulating cathepsin B expression and microglia autophagy, thereby contributing to long-term protection.

#### Others

Moderate physical activity possesses a multitude of neuroprotective effects. Physical exercise favors stroke prognosis by mitigation of inflammatory responses, suppression of neuro-excitotoxicity, attenuation of apoptosis, enhancement of angiogenesis, neurogenesis and even synaptogenesis, improvements in cerebral blood flow, and enhancement of brain microvascular integrity (Zhang Q. et al., 2014). Following global cerebral ischemia in Wistar rats, post-ischemic exercise has been shown to reverse the ischemia-induced increase of the area occupied by microglia and to modulate astrocyte and microglia in the hippocampal dentate gyrus (Lovatel et al., 2014).

Acupuncture, a traditional Chinese medical treatment, includes manual acupuncture, electroacupuncture, and moxibustion (Hou et al., 2021). Several studies demonstrated that acupuncture has the potential to be neuroprotective and immunomodulatory in ischemic stroke through microglia targeting, such as inhibition of microglia-mediated neuroinflammation and suppression of NF-κB-mediated microglia activation (Liu et al., 2016; Liu R. et al., 2020).

Repetitive transcranial magnetic stimulation (rTMS) has been widely used in the rehabilitation of stroke patients, due to its clinical advantages of non-invasiveness and safety. However, the biological mechanism underlying its positive effects are complicated and include the rebuilding of brain networks, modulation of the transmission of neurotransmitters, amelioration of cerebral metabolism and circulation, and regulation of brain excitability (Luo et al., 2022). Several studies showed that rTMS can reduce the production of proinflammatory mediators and suppress microglia activation (Yang et al., 2020; Zong et al., 2020). Luo et al. (2022) found that long-term rTMS enhanced neurogenesis, suppressed apoptosis, and regulated inflammation by downregulating the secretion of NF-κB and STAT6 and inducing the polarization of anti-inflammatory microglia.

Red or near-infrared light can alleviate oxidant activity and inflammatory responses, control microglial activities and bioenergetic metabolism, promote neurogenesis and synaptogenesis, and improve blood flow (Hamblin, 2018; Salehpour et al., 2018). Vogel et al. (2021) reported that transcranial low-level laser-induced photobiomodulation can inhibit microglia activation, attenuate neuroinflammation, enhance glial fibrillary acidic protein (GFAP) expression, and eventually reduce the ischemic lesion volume.

Despite the promise of the abovementioned therapeutics, a history of discrepancies between preclinical and clinical studies imply that we should be cautious in analyzing preclinical outcomes. Improving and refining experimental models may help us determine whether these methods are indeed useful in the treatment of CNS pathologies.

## Conclusion and Perspectives

Microglia are brain-resident macrophages that can sense even small disruptions to homeostasis and are rapidly activated *via* changes in anatomical structure and physiological function. Microglia communicate with neurons, astrocytes, oligodendrocytes, and endothelial cells during and after ischemic stroke. By assuming different degrees of polarization, activated microglia are considered a double-edged sword in the battle for neurological recovery. There is an urgent need to establish an effective clinical strategy to modulate microglia activation and drive their polarization to the most protective phenotype at various stages of ischemic stroke.

The current state of knowledge on microglia phenotypes offers potential new avenues for CNS repair after acute injury. Based on our observations, many chemicals and biomolecules can induce M1-like microglia; however, only a portion of microglia are converted into M1-like microglia, while others remain M2-like or are not activated. Similarly, only a portion of microglia are converted into M2-like microglia by M2-like inducers. Whether there is a dominant molecule/pathway that uniformly shifts microglia phenotype requires further investigation. A combination of multiple new approaches such as cell-targeted deletion, single cell RNA sequencing, epigenetics, and high throughput screening may provide additional information on how to uniformly shift microglia. It will also be important to learn how to modulate a specific beneficial state without switching to the opposite phenotype. As M2-like microglia are critical for long-term recovery after ischemic stroke, further research is warranted on how to maintain more microglia for longer durations in the M2-like phenotype. The action of phenotype inducers is likely to be timing and dose-dependent, rather than a simple all-or-none process. Thus, the identification of extracellular molecular and intracellular key signals that control phenotypic switches will be important in identifying an appropriate clinical treatment regimen. In addition, the roles of proinflammatory and anti-inflammatory microglia are not necessarily destructive *vs*. beneficial, respectively. For example, M1-like microglia may drive the clearance of cellular debris and contribute to synaptic remodeling after stroke, while long-term maintenance of M2-like microglia may suppress immune defenses or induce tumorigenesis. It may also be difficult to replicate the complex temporal and spatial effects of microglia with exogenous delivery of a single factor. Thus, it is important to continue to explore microglial signaling pathways to identify multiple therapeutic targets and understand the morphological and functional evolution of microglia over time after the onset of brain injury.

## Author Contributions

YW and GC: study concept and design. YW: drafting of the manuscript. RL and GC: critical revision of the manuscript for important intellectual content. All authors contributed to the article and approved the submitted version.

## Funding

GC was supported by RF1NS117509 from Office of Extramural Research, National Institute of Health and Merit Review Grants BX003923 from the U. S. Department of Veterans Affairs.
